# Hemophilia of orbit

**DOI:** 10.4103/0974-620X.53039

**Published:** 2009

**Authors:** Gunaseelan Karunanithi, Pooja Sethi, Sathyanarayana K. Reddy, Vivekanandam

**Affiliations:** Department of Radiotherapy, Jawaharlal Institute of Postgraduate Medical Education and Research, Puducherry, India

**Keywords:** Hemophilia, orbit, radiotherapy, pseudotumor

## Abstract

Hemophilic pseudotumor is an uncommon complication of factor VIII and IX deficiencies in the coagulation cascade and occurs in a wide spectrum of bones and soft tissues. We present a six-year-old boy with hemophilic pseudotumor localized in the right orbit. He showed a favorable response to radiation therapy after unsuccessful treatment with factor VIII replacement therapy with no recurrence till eight months. Radiotherapy in the treatment of pseudotumors in hemophiliacs should be strongly considered, particularly in severely affected patients who do not respond to conservative therapy.

## Introduction

Hemophilia A and B are the blood clotting disorders caused by mutation of the factor VIII and factor IX genes respectively, which lead to defective synthesis or synthesis of dysfunctional factor VIII or IX. Hemophilia A is more common than hemophilia B. Inheritance is X-linked recessive; hence, males are affected while females are carriers. A hemophilic pseudotumor is an encapsulated, chronic, slowly expanding hematoma, due to recurrent hemorrhage; and is seen in 1–2% patients with severe coagulative disorder (less than 1% of normal factor VIII activity). It usually occurs in soft tissues, muscles, tendons and subperiosteal part of bones. The tumor enlarges slowly, develops a fibrous capsule, and can destroy underlying tissues by progressive necrosis.[1] We describe a six-year-old boy with hemophilia A who presented with a hemophilic pseudotumor in the right orbit, which was resistant to conservative treatment and finally responded to low dose external beam radiation. The role of radiotherapy in the treatment of hemophilic pseudotumor is reviewed.

## Case Report

**Figures 1 and 2:**
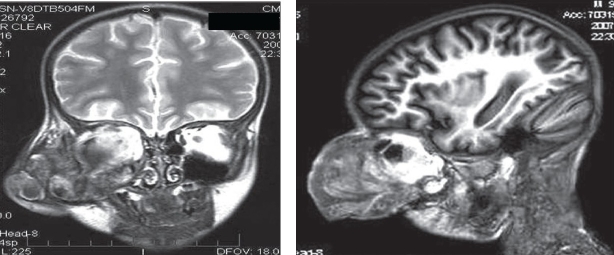
(T2 W-MRI images) Well-defined enhancing irregular mass and hyperintense periphery in the retrobulbar space

**Figures 3 and 4:**
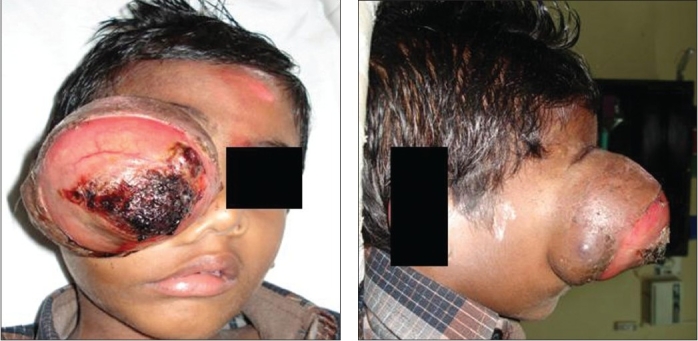
Clinical picture of patient posttraumatic (at four weeks) hemophilic pseudotumor orbit

A six-year-old boy diagnosed with hemophilia A since the age of 3 years, sustained blunt trauma to the right side of the face. He attended our institute one week after trauma with a painful right eye with periorbital oedema, marked proptosis (nearly 8 cm), hyphema, and visual acuity of no light perception. Because of total hyphema and exposure keratopathy, fundus examination was not feasible. Intraocular pressure was more than 25 mm Hg. Due to rapidly progressing unilateral proptosis of eye, differential diagnosis of retrobulbar hemmorhage or rhabdomyosaroma was made. Orbital magnetic resonance imaging (MRI) was performed immediately, which showed a large 77 x 66 x 68 mm, well-defined, enhancing, irregular mass arising from the right orbit, with heterogenous signal intensity, and a rim of peripheral hyperintensity on short TR sequences with posterior fluid levels of layering, suggestive of hemorrhage. The right globe was not seen separately [Figures 1 and 2]. These characteristic findings in a patient with severe coagulation disorder led to the diagnosis of hemophilic pseudotumor of the right orbit. The patient was started on conservative management with 15,000 units of factor VIII; 18 units of cryoprecipitates; 11 fresh frozen plasma; four packed red blood cells; and two whole blood infusions. Despite these measures, the proptosis increased slowly in the initial 10 days and then progressed rapidly in the next two weeks [Figures 3 and 4]. His antihemophilic factor inhibitor titre was 1536 Bethesda units, and Factor VIII assay was <1%.

**Figures 5 and 6:**
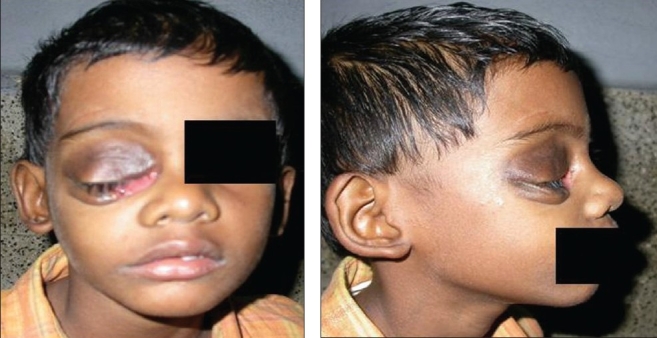
Clinical picture 12 weeks after completion of radiotherapy

With conservative management, the proptosis kept on increasing. Surgical intervention was contraindicated since the child had circulating antihemophilic inhibitor. He was therefore treated with low dose external beam radiation using 6 MV linear accelerator. He received a total dose of 900 cGy over 5 fractions in 5 days, by direct anterior single field of size 8 x 8 cm. Pain started to subside on third day of radiotherapy and completely ceased by the tenth day. The size of pseudotumor gradually decreased, and proptosis completely disappeared by four weeks after completion of radiotherapy. By the end of 12 weeks, the tumor had completely resolved [Figures 5 and 6].

The patient has been under follow-up for eight months since treatment. During this time no evidence of tumor recurrence was observed.

## Discussion

Hemophilic pseudotumor is an uncommon complication of factor VIII and IX deficiencies in the coagulation cascade and occurs in a wide spectrum of bones and soft tissues. A pseudotumor consists of chronically encapsulated blood collection due to recurrent extraarticular hemorrhage in either bone or soft tissues. As the swelling progresses, increasing pressure leads to the slow destruction of adjacent structures.[2]

Diagnosing a hemophilic pseudotumor with invasive techniques such as, aspiration and biopsy are not advisable due to increased risk of complications (hemorrhage, infection). Imaging techniques of which MRI is preferred, allows recognition of blood products in various stages of evolution.[3] Ultrasonography (USG) shows a central anechoic region with increased echoes behind the lesion due to enclosed fluid in the pseudotumor. Computed Tomography (CT) identifies the thick pseudocapsule, but cannot differentiate a hematoma from a chronic abscess. MRI signals vary with the age of blood progressing from being isointense with muscle in the first week on T1-W images, and low signal intensity on T2-W images. T1-W signal intensity subsequently increases. After the first week, the peripheral portion becomes hyperintense on both T1- and T2-weighted images. The wall tends to be hypointense because it contains hemosiderin.[4] CT is particularly helpful in the evaluation of bone, whereas MRI is superior to CT for delineating soft tissue and intramedullary spaces.

The initial treatment of a hemophilic pseudotumor is conservative with clotting factor replacement.[3] Radiotherapy with or without replacement therapy has shown promising results as an alternative to a more mutilating surgery or a contraindicated surgery in the treatment of hemophilic pseudotumor, resistant to conservative treatment. Radiation results in: a) endarteritis in an acute bleeding hematoma; b) direct injury of small vessels causing fibrosis and healing; and c) stimulation of fibroblasts resulting in fibrosis.[5] Kapoor *et al*. reviewed 21 patients with hemophilic pseudotumors receiving radiotherapy, with or without factor VIII replacement. Majority of patients had long bone involvement followed by small bones;[6] only one case involved the orbit. Radiotherapy with 7.5 Gy external beam radiotherapy was reported by Meyers *et al*.[7] There has been considerable variation in the literature in radiotherapy dose. Also, the doses, as low as 600 cGy to as high as 2350 cGy, with or without factor VIII replacement, have shown good response with complete resolution of lesions.[6] Even though, no standard radiation dose and fractionation schedule exists in the management of hemophilic pseudotumors, radiation therapy should be tried in cases where surgery is not feasible. For fear of life threatening complications of surgery in this child, radiation was preferred, and a low dose radiation was given. The tumor responded well to radiation. Vision however, remained at the no light perception level.

We conclude that low dose radiotherapy can be a safe and effective mode of the treatment in orbital hemophilic pseudotumor, especially in patients not responding to conservative management.
